# Mothers with and without bipolar disorder and their infants: group differences in mother-infant interaction patterns at three months postpartum

**DOI:** 10.1186/s12888-019-2275-4

**Published:** 2019-09-18

**Authors:** Teija M. S. Anke, Kari Slinning, Vibeke Moe, Cathrine Brunborg, Torill S. Siqveland, Dag Vegard Skjelstad

**Affiliations:** 1 Vestre Viken Hospital Trust Division of Mental Health and Addiction, Drammen, Norway; 2 Centre for Child and Adolescent Mental Health, Eastern and Southern Norway, R.BUP, Oslo, Norway; 3 Department of Psychology, University of Oslo, Oslo, Norway; 4 Oslo Centre for Biostatistics and Epidemiology, Research Support Services, Oslo University Hospital, Ullevål, Oslo, Norway; 5 Oslo University Hospital, Ullevål, Oslo, Norway

**Keywords:** Bipolar disorder, Postpartum, Mother-infant interaction, Affective symptoms, Dyadic coordination

## Abstract

**Background:**

Women with bipolar disorder (BD) have a high risk of illness relapse postpartum. The risk coincides with the period when mother-infant interactions are evolving. We compared mother-infant interactions in dyads where the mothers have BD with dyads where the mothers have no mental disorder. The association between concurrent affective symptoms of BD mothers and interaction quality was investigated.

**Methods:**

Twenty-six women with BD and 30 comparison women with infants were included. The Parent-Child Early Relational Assessment (PCERA) was used to assess maternal behaviour, infant behaviour and dyadic coordination in interactions at 3 months postpartum. The Inventory of Depressive Symptomatology and Young Mania Rating Scale were used to assess affective symptoms of BD mothers at the time of interaction.

**Results:**

There were significant group differences with medium to large effect sizes (0.73–1.32) on five of six subscales within the three interactional domains. Most interactional concerns were identified in dyadic coordination. No significant associations were found between maternal symptom load and interaction quality within the BD sample. Forty-six percent of the BD mothers experienced a mood episode within 0–3 months postpartum.

**Conclusions:**

The present study identified challenges for mothers with BD and their infants in “finding” each other in interaction at 3 months postpartum. If sustained, this interaction pattern may have a long-term impact on children’s development. We suggest interventions specifically focusing on sensitising and supporting mothers to read infants’ cues on a micro-level. This may help them to respond contingently and improve dyadic coordination and synchronicity.

## Background

The quality of mother-infant interaction is suggested as an important environmental mediator between perinatal mental disorder in mothers and infant development [1]. Emerging evidence implies that there may be disorder-specific deviations in interaction patterns [1–7]. To date, there has been little research on mother-infant interactions in the context of maternal bipolar disorder (BD).

For women with BD, childbirth and the postpartum period yield an increased risk of illness relapse compared with non-postpartum periods [8–11]. Estimations suggest an approximate one in five risk for a severe illness relapse [9–11] and one in two deliveries resulting in any affective episode [10, 11]. Thus, a high-risk period of illness relapse coincides with the period when mother-infant interaction patterns are evolving.

Within a dyadic system approach, interaction is defined as a bi-directional and dynamic process consisting of three domains: 1) parental contribution, 2) infant contribution, and 3) dyadic coordination [12, 13]. Attentiveness, sensitivity to infant cues and contingent responsiveness, are underscored as crucial parental components [13–15]. Potentially, BD symptoms with marked alterations in affect, energy, activity and cognition may impact these maternal behaviours. As a group, infants of mothers with BD have been linked to an increased risk for negative birth outcomes, such as neonatal readmissions and morbidity, small for gestational age (SGA <2nd-3rd percentile), low birth weight (< 2500 g) and prematurity [16]. Furthermore, preliminary findings suggest that the infants have disruptions in their physiological stress responsivity and regulation, which may imply an increased susceptibility to stressors [17]. Thus, there are risk factors that may affect interactional contributions on behalf of both the mother and infant, as well as potentially indicating the infants’ increased need for sensitive care. The third domain, dyadic coordination, concerns whether the interactional behaviours of mother and infant are mutual and synchronised. Based on empirical findings, dyadic coordination is considered particularly indicative for child development [12, 13, 18, 19].

To the best of our knowledge, there are only three studies where the different domains of mother-infant interactions have been investigated within the first year postpartum, when the mother has BD. Maternal interaction behaviour was studied among mothers with BD who were admitted to a Mother and Baby Unit (MBU) within 1–36 weeks postpartum [20]. MBUs are specialised perinatal psychiatric units, where women with mental illness episodes are receiving mental health care and support in their relationship with their infant. Mothers with BD and schizophrenia deviated more from the normal range in their interaction behaviour, than mothers with unipolar depression [20]. Ten mothers with BD and nine with unipolar depression were reassessed at 12 months postpartum [21]. All had recovered from their prior postpartum episodes, but the mothers in the study group were evaluated as less sensitive and appropriate in their behaviour, with more negative affect than healthy controls. However, BD mothers showed significantly more affectionate talk to their infants than mothers with unipolar depression. Furthermore, the infants in the study group displayed a non-significant trend to be less expressive than controls [21]. When comparing maternal sensitivity and mother-infant reciprocity at 12 months postpartum among three groups of mothers (bipolar depression, unipolar depression and non-depression), mothers with bipolar depression obtained poorer scores than the other two groups. However, the differences were not significant [22].

With a scarce evidence base indicating difficulties, further research is needed, not least because interaction quality as a suggested mediator between maternal mental disorder and infant development is potentially modifiable [1, 16, 23].

In particular, we suggest that studies should include all three interactional domains to provide a comprehensive picture of mother-infant interactions. Furthermore, studies in early postpartum are needed. The majority of birth-related affective episodes develop before 6 months postpartum [8, 10], with the most severe (i.e., postpartum psychosis) often within 4 weeks after childbirth [10]. Moreover, two major biobehavioural shifts in infant development occur before 12 months, with the first appearing at 2–3 months. At this age, the infant’s capability of participating in synchronous interactions is greatly enhanced, with emerging memory and anticipation of recurring interaction patterns [19, 24]. Early detection of possible deviations is also important to lessen the negative impact on development [24] and possibly the risk of subsequent mental disorders, including BD.

Finally, we know little about how variations in maternal symptom load may affect interaction, apart from one study where illness relapse requiring hospitalisation was associated with clear deviations in maternal behaviour [20].

In the present study, we compared mother-infant interactions in dyads where the mothers had BD with dyads where the mothers had no mental disorder. All three interactional domains (maternal behaviour, infant behaviour and dyadic coordination) were assessed at 3 months postpartum. As suggested by the aforementioned studies on maternal BD [20–22] and other maternal mental disorders [1–7], we anticipated more concerns in the mother-infant interactions of the BD sample than in the comparison group. We expected differences in all three interactional domains. Furthermore, we hypothesised that concurrent symptom load in the BD sample was negatively associated with interaction quality.

## Methods

### Design

The study is part of a Norwegian prospective investigation. Infant families where the mother has BD are studied from pregnancy to 12 months postpartum, with data collection at four time points.

### Recruitment procedures and subjects

#### BD sample

Inclusion criteria for the study were women with a BD I or II diagnosis, with a cohabitating partner, and who were either pregnant or had recently given birth (within 3 months). Because of the main aims of the larger investigation, their partner had to be willing to participate. The exclusion criteria were parental substance abuse, multi-childbirth, premature birth < 35 weeks, or an infant with a known serious medical condition or syndrome. All eligible participants who consented to participate were included.

We provided oral and written information about the study to health professionals at mental health outpatient clinics and wards, infant mental health teams at child mental health services, community well-baby clinics, pregnancy care and maternity wards. We also informed about the study through the website of the national BD association and at group psychoeducation courses for patients with BD [25].

Recruitment took place between September 2014 and July 2016. Most participants (58%) were recruited from the geographic area of Vestre Viken Hospital Trust. The remaining participants were recruited from nearby counties in the south-eastern part of Norway.

All participants gave oral and written consent on behalf of themselves and their infant at an information meeting with TA (first author). The consent allowed TA to have the women’s clinical BD diagnosis verified from their specialist mental health records and/or by contacting their specialist mental health professional. In addition, TA assessed the BD diagnosis using a semi-structured interview and discussed the diagnosis with the last author when needed.

Thirty-five women were interested in participating. In three cases, the woman’s partner declined to participate. Three women were assessed not to have BD. One woman did not respond to TA’s calls for setting up a meeting, and two women changed their mind. Thus, the final sample comprised 26 women diagnosed with BD. We have no record of the number of eligible women who declined to participate when informed by collaborating health professionals.

#### Non-clinical sample

Data for comparison of mother-infant interactions were included from another Norwegian study. The comparison group consisted of 30 mother-infant dyads, recruited from local well-baby clinics in Oslo, Norway, between December 2004 – January 2009 [26]. Inclusion criteria for the comparison group were being pregnant and having no substance abuse or mental health problems. The mothers’ mental health status was investigated in pregnancy with European Addiction Severity Index [27], Millon’s Clinical Multiaxal Inventory-III [28] and Hopkins Symptom Check List, SCL-25 [29]. At 3 months postpartum, the Edinburgh Postnatal Depression Scale (EPDS) [30] was administered to assess the presence of depressive symptoms. The maximum score on the EPDS is 30, and a score ≥ 10 indicates a risk for postpartum depression. The EPDS mean score in the comparison group was 3.16 (SD 3.20, range 0–12), corresponding to a low depression risk. All women in the comparison group also had a cohabitating partner. All participants gave oral and written consent on behalf of themselves and their infant at the time of enrolment.

### Assessments

#### Mother-infant interaction at three months postpartum

The same interaction situation and method of assessment were used for both samples. Mothers and infants were video-recorded in a 5-min free-play interaction situation. The recordings of the BD sample were performed at the participants’ home (*n* = 25) or at an outpatient clinic (*n* = 1). The latter was the case for all recordings of the comparison group. The mothers were asked to interact with their infant as they were used to and as they pleased, with optional use of toys. At the end of the video-recording session, the mothers in both samples subjectively evaluated the representativeness of the play interaction. All mothers, except one in the BD sample who felt awkward because of the video-recording, regarded the play interaction as representative.

The mother-infant interactions were analysed using the Parent-Child Early Relational Assessment (PCERA) [31]. It is a standardised assessment method that has demonstrated good content and factor validity, as well as discriminant validity between clinical and non-clinical groups [32, 33]. The PCERA aims to examine strengths and concerns in parental and infant components and in their dyadic pattern. It contains 65 behavioural, affective and communicative variables. These are operationalised in a manual and rated numerically based on observed frequency, duration and intensity. Rating is performed on a five-point Likert scale. The five points are categorised into three areas of concern/strength according to PCERA: (1, 2) area of concern, (3) area of some concern and (4, 5) area of strength [31].

In the present study, all interactions in the BD sample were rated by an independent certified main coder. A second independent certified coder double-rated a random selection of 31% of the interactions for calculation of inter-rater reliability. A good inter-rater reliability was found using absolute agreement on ratings (intra-class correlation 0.75). The coders were aware of the women’s BD diagnosis but were blinded to all other information.

In the study from which the comparison group data were derived, two independent experienced coders rated the interactions. The main coder in the present study was one of the coders. Both coders double-rated 20% of randomly selected interactions, and inter-rater reliability was calculated using categorical agreement (1-2, 3, 4-5). Intra-class correlation varied between 0.80 and 0.97 for the different subscales used in the study [26]. All information, including group status (no mental health problems vs. substance abuse or mental health problems), was unknown to the coders.

#### Maternal symptom load at three months postpartum in the BD sample

Assessments of symptom load were conducted at the same time-point as the interaction session. Depressive symptoms were assessed with the Inventory of Depressive Symptomatology (IDS) [34] and hypomanic/manic symptoms with the Young Mania Rating Scale (YMRS) [35].

We collected information about postpartum affective symptoms and episodes at least once at 1–2 months postpartum, either by direct contact with the mothers and/or their respective specialist mental health professionals. Furthermore, this was examined retrospectively in an interview with all women at 3 months, in conjunction with the interaction session and assessment with IDS and YMRS. Two mothers joined the study at 3 months postpartum and had not been in contact with any mental health care system postpartum.

### Statistical analyses

Demographic and clinical data are presented as either proportions or means with their standard deviations (SD) and range. When conducting analyses on interaction data, PCERA variables were organised into six subscales according to the manual, with two maternal, two infant and two dyadic subscales [31]. Subscales are used since not all variables in the PCERA are applicable for all child ages. In the present study, subscales appropriate for infant age 3–4 months were chosen. However, two variables from the original infant subscales were excluded. “Quality of exploratory play” was excluded on recommendation from the coders, since this is a difficult variable to rate on infant age 3 months. “Consolability/soothability” is not possible to rate if no need for soothing occurs during the interaction. The final subscales contained 25 maternal, 17 infant and 8 dyadic variables (see Table 1).

**Table 1:** PCERA maternal, infant and dyadic subscales utilised, with Cronbach’s alpha for both samples

Subscale	Variables included in subscales	Cronbach’s alpha BD sample vs. Non-clinical sample	
S1-Maternal positive affective involvement, sensitivity and responsiveness	2)^a^ Expressive, non-flat tone of voice 3) Warm, kind tone of voice 4) Expressed positive affect 7) Lack of depression, withdrawn mood 9) Enthusiastic mood 12) Enjoyment, pleasure 13) Positive physical contact 15) Visual contact 16) Amount of verbalisation 17) Quality of verbalisation 18) Social initiative 19) Contingent responsivity 22) Sensitivity, reads cues and responds 23) Connectedness 24) Mirroring 26) Creativity	0.92	0.96
S2-Maternal negative affect and behaviour	1) Angry, hostile tone of voice 3) Warm, kind tone of voice 5) Expressed negative affect 6) Angry, hostile mood 11) Displeasure 14) Negative physical contact 16) Amount of verbalisation 19) Contingent responsivity 21) Lack of structuring and mediating 22) Lack of sensitivity and responsivity 25) Rigidity 27) Intrusiveness 28) Inconsistency/unpredictability	0.88	0.93
S3-Infant positive affect, communicative and social skills	30) Expressed positive affect 32) Happy, pleasant 33) Apathetic, withdrawn 38) Alertness 39) Social initiative 40) Social responsiveness 47) Robustness 55) Visual contact 56) Communicative competence 57) Readability	0.96	0.94
S4-Infant dysregulation and irritability	31) Expressed negative affect 34) Anxiety 35) Irritable, angry 37) Emotional lability 41) Avoiding/averting 46) Attentional abilities 50) Self-regulation, organisation	0.80	0.87
S5-Dyadic mutuality and reciprocity	59) No flat, empty, constricted 61) Enthusiasm, joie de vivre 63) Reciprocity	0.96	0.88
S6-Dyadic tension	58) Anger, hostility 60) Tension, anxiety 62) No joint attention, activity 64) Disorganisation 65) State dissimilarity	0.86	0.90

^a^Variable number in manual [31]

Group differences, using PCERA mean scores on the subscales, were analysed using independent samples t-tests. The chi-square test for contingency tables or Fisher’s exact test was used to detect associations between categorical variables and BD vs. the non-clinical sample. Correlation analyses were performed separately for BD and non-clinical samples using Pearson’s correlation coefficient (r).

To identify possible confounders, we studied all variables that could influence the outcome known from the literature. Possible confounding factors investigated were maternal age, education, work participation, parity, infant gestational age, infant gender, birth weight and infant exact age at the interaction session. Only variables with significant relationships with both the exposure (BD vs. non-clinical) and the outcome variables (PCERA maternal, infant and dyadic subscales) were considered as possible confounders and included in the multiple linear regression analysis.

Within the BD sample, we conducted an independent samples t-test to investigate whether infant exposure to maternal BD medication in pregnancy and postpartum was associated with PCERA mean scores.

Pearson correlation analyses and linear regression analyses were used to examine the association between maternal symptom load as a continuous variable and the outcome variables. Additionally, we dichotomised symptom scores to obtain categorical variables. An IDS score of 20 and a YMRS score of 14 were used as cut-offs between “low” and “high” symptom loads. According to the IDS scale, a score of 21 is the cut-off between mild and moderate symptom load [34]. Based on our data, we defined 20 as a cut-off between “low” and “high” symptom load, in order to obtain a fairly equal distribution of the BD participants in two groups. This is purposeful for statistical analysis with a small sample. The same logic was applied when deciding a score of 14 as cut-off on YMRS. According to the YMRS, the mean score of patients who clinically are assessed as hypomanic/lightly manic is 13 [35]. Differences in PCERA mean scores between “low” and “high” symptom load were analysed with independent samples t-tests.

Data regarding the presence of symptoms and affective episodes 0–3 months postpartum are used only for descriptive purposes. The total data were complex and would result in too many variables for statistical analysis of correlation in a small sample.

Overall, a significance level of 0.05 was applied. Effect sizes were calculated by Cohen’s *d*. Small effect sizes were defined as 0.20, medium as 0.50 and large as 0.80 and higher [36]. The internal consistency of the subscales was examined using Cronbach’s α. An α value > 0.70 is considered satisfactory, and α values ≥0.90 are considered excellent.

The proportions of the BD sample and the non-clinical sample were organised into three areas of concern/strength (scores 1–2, 3, 4–5) to show the distribution of interaction quality.

Data were analysed using the IBM SPSS statistics for Windows version 23 (Armonk, NY, USA: IBM Corp).

## Results

### Sample characteristics

The maternal and infant characteristics of the two samples are presented in Table 2.

**Table 2:** Characteristics of mothers and infants in the BD and non-clinical sample

Variable	BD sample *N* = 26		Non-clinical sample *N* = 30		*p*-value
Maternal age at inclusion (years) mean ± sd; range	30.5 ± 4.6; 22–37		33.3 ± 5.0; 27–44		0.04
	n	%	n	%	
Primary diagnosis					Not applicable
BD I	7	27	0		
BD II	19	73	0		
Parity					0.32
Primiparous	13	50	20	67	
Multiparous	13	50	10	33	
Completed education					< 0.001
Primary school	8	31	1	3	
Secondary school	5	19	5	17	
Bachelor’s degree	11	42	8	27	
Master’s degree	2	8	16	53	
Work participation when not pregnant					0.002
Working full-time	12	46	23	77	
Working part-time +/− receiving benefits	4	15	2	7	
Receiving benefits only	8	31	0		
Unemployed	1	4	1	3	
School	1	4	4	13	
Infant gender					0.91
Girl	10	38	12	40	
Boy	16	62	18	60	
Infant exposure to BD medication					Not applicable
In pregnancy	17	65	0		
In pregnancy + 2–3 months postpartum	10	38	0		
Infant birth weight (gram)	3632 ± 507;		3720 ± 434;		0.49
mean ± sd; range	2905–5085		2911–4715		
Infant gestational age (months)	39.5 ± 1.2;		40 ± 1.2;		0.13
mean ± sd; range	37.2–41.6		37–42		

The non-clinical sample had significantly higher maternal age, education, and work participation than the BD sample. Gestational age and birth weight were within the normal range for both infant samples, and there were no significant group differences. A majority of the infants in the BD sample (65%) were exposed to BD medication in pregnancy. As some mothers chose not to breastfeed when on medication, the proportion of infants exposed both in pregnancy and postpartum was lower (38%).

### Mother-infant interaction

Table 3 demonstrates that there were significant group differences with medium to large effect sizes (Cohen’s *d* 0.73–1.32) on all subscales, except on subscale 4, “Infant dysregulation and irritability”.

**Table 3:** Interaction score comparisons (mean) between BD sample and non-clinical sample on PCERA subscales

Subscale	BD sample Mean (sd) 95% CI	Non-clinical sample Mean (sd) 95% CI	Mean difference 95% CI	Sign./ Adjusted sign.	Cohen’s *d*
S1-Maternal positive affective involvement, sensitivity and responsiveness	3.7 (0.43)	4.1 (0.63)	−0.40 (−0.70 to −0.10)	0.01^*^/0.04^*^	0.73
	3.5–3.8	3.8–4.3			
S2-Maternal negative affect and behaviour	4.0 (0.39)	4.4 (0.54)	−0.38 (− 0.63 to − 0.12)	0.004^*^/0.03^*^	0.81
	3.8–4.1	4.2–4.6			
S3-Infant positive affect, communicative and social skills	3.2 (0.71)	3.7 (0.70)	−0.51 (− 0.89 to − 0.13)	0.01^*^	0.73
	2.9–3.5	3.5–4.0			
S4-Infant dysregulation and irritability	4.0 (0.46)	4.0 (0.63)	−0.003 (− 0.29 to 0.29)	0.98	0.01
	3.9–4.2	3.8–4.3			
S5-Dyadic mutuality and reciprocity	2.6 (0.83)	3.7 (0.82)	−1.08 (−1.53 to −0.64)	< 0.001^*^	1.32
	2.3–3.0	3.4–4.0			
S6-Dyadic tension	3.6 (0.52)	4.1 (0.71)	−0.46 (− 0.80 to − 0.13)	0.01^*^/0.04^*^	0.74
	3.4–3.8	3.8–4.4			

**Fig. 1:**
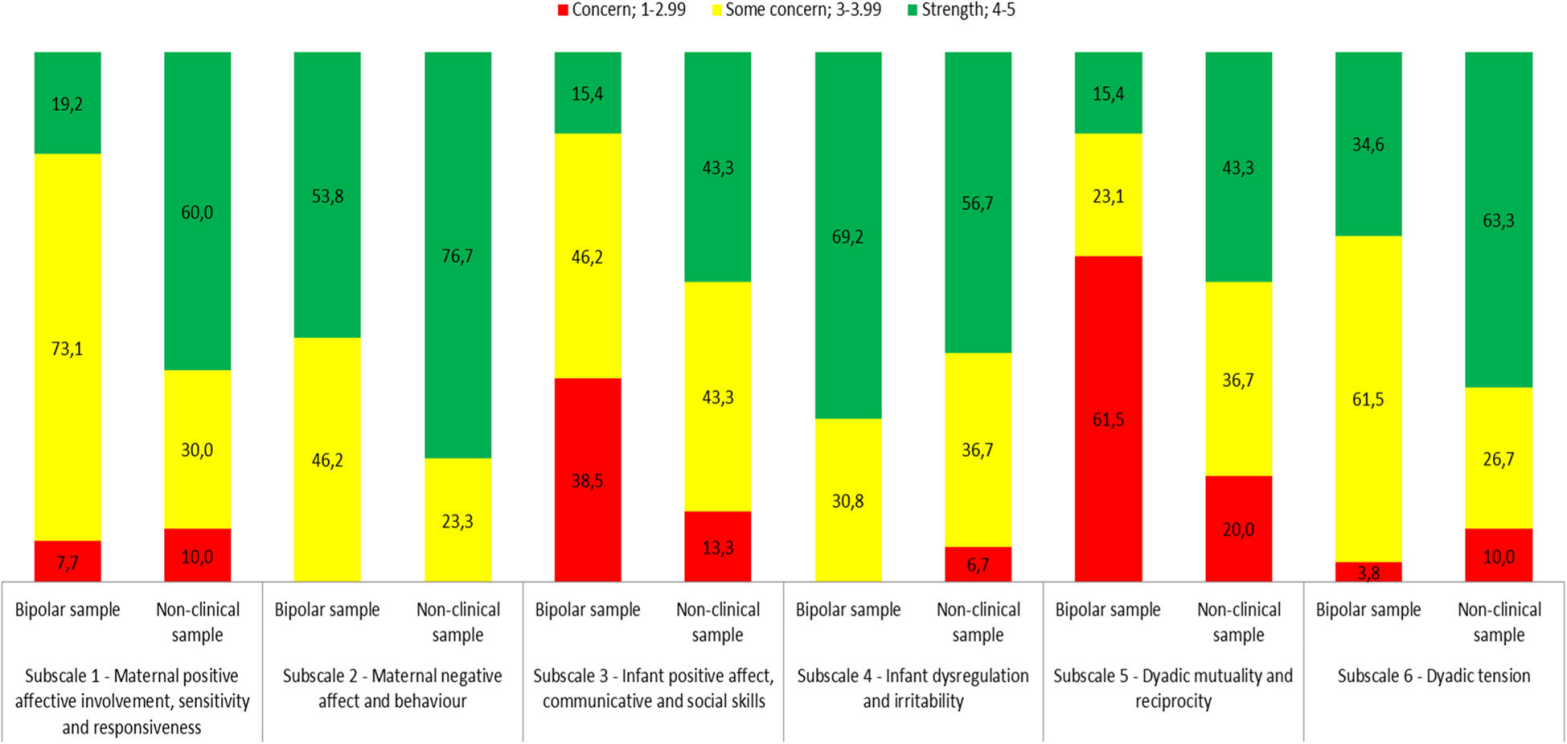
Percentages of BD sample and non-clinical sample in three concern/strength categories according to PCERA

Figure 1 shows the distribution of the BD and the non-clinical sample on the three categories of concern/strength for each subscale.

The proportion of BD and comparison dyads displaying interaction behaviour within the area of strength varied between 15.4–69.2% and 43.3–76.7%, respectively, depending on the subscale.

#### Maternal subscales

The BD sample scored significantly lower on subscale 1, “Maternal positive affective involvement, sensitivity and responsiveness”, than the non-clinical sample (mean difference (∆_mean_): − 0.40, 95% confidence interval (CI): − 0.70 to − 0.10). In multiple linear regression analysis, this association remained significant after controlling for the confounding effect of maternal age (adjusted ∆_mean_: − 0.32, 95% CI: − 0.62 to − 0.02). No other confounding variables were found.

On subscale 1, a large majority of mothers in the BD sample (80.8% vs. 40%) were in the some concern – concern range (Fig. 1). Inspection of the 16 individual variables included in subscale 1 revealed that the differences between the groups were mainly identified on the variables “social initiative”, “quality of verbalisation”, “sensitivity, reads cues and responds”, “contingent responsivity” and “mirroring”, that is, on the variables that entail involvement. The BD sample did not differ from the comparison group on the variables “expressed positive affect”, “visual contact” and “amount of verbalisation”.

On subscale 2, “Maternal negative affect and behaviour”, the BD sample scored significantly lower than the comparison group (∆_mean_: − 0.38, 95% CI: − 0.63 to − 0.12). This association remained significant after adjusting for maternal age. Approximately half of the BD sample (46.2% vs. 23.3%) was in the some concern range on subscale 2, whereas none of the participants were in the concern range (Fig. 1). Considering the 13 individual variables included in subscale 2, the group differences were primarily on the same and similar variables as on subscale 1, “contingent responsivity”, “lack of structuring and mediating” and “lack of sensitivity and responsiveness”. In both groups, there were few signs of overt negative affect, anger and hostility.

#### Infant subscales

The BD sample had a significantly lower score on subscale 3, “Infant positive affect, communicative and social skills”, than the non-clinical sample (∆_mean_: − 0.51, 95% CI: − 0.89 to − 0.13). No confounding factors were found for this association. Figure 1 displays that 84.7% (vs. 56.6%) of the infants in the BD sample were in the some concern - concern range. The largest group difference was found in concern scores (BD 38.5% vs. 13.3%). Inspection of the 10 individual variables on subscale 3 identified less optimal scores for the infants in the BD sample on “expressed positive affect”, “happy, pleasant”, “apathetic, withdrawn”, “communicative competence” and “social responsiveness”.

No mean difference on subscale 4, “Infant dysregulation and irritability”, was found between the BD and non-clinical samples (∆_mean_: − 0.003, 95% CI: − 0.29 to 0.29) after adjusting for the confounding effect of maternal age. A higher percentage (43.4%) of the infants in the non-clinical sample had scores in the some concern - concern range on subscale 4 compared with the BD sample (30.8%) (Fig. 1); however, the difference was not significant.

#### Dyadic subscales

The largest mean difference between the groups was found on subscale 5, “Dyadic mutuality and reciprocity”, showing that the BD sample on average scored more than 1 point lower than the non-clinical sample (∆_mean_: − 1.08, 95% CI: − 1.53 to − 0.64). No confounding factors were found. A total of 61.5% of the dyads in the BD sample were in the concern range (vs. 20%), and 23.1% were in the some concern range (vs. 36.7%) (Fig. 1). There were large group differences on all three variables on the subscale, with the BD dyads having less optimal scores on “flat, empty, constricted”, “enthusiasm, joie de vivre” and “reciprocity”.

The overall mean score was significantly lower in the BD sample than in the non-clinical sample on subscale 6, “Dyadic tension”, (∆_mean_: − 0.46, 95% CI: − 0.80 to − 0.13). This association remained significant after adjusting for the confounding effect of maternal age and work participation. A total of 65.3% (vs. 36.7%) of the BD sample were in the some concern - concern range (Fig. 1). Inspection of the 5 individual variables on the subscale revealed group differences on the variables “no joint attention, activity”, “disorganisation” and “state dissimilarity”. None of the groups were characterised by dyadic “anger, hostility” or “tension, anxiety”.

Finally, no significant associations were found between infant medication exposure and mean scores on any of the subscales in the BD sample (Additional file 1). Though, the association was of medium effect size (Cohen’s *d* 0.53) on subscale 2.

### Association between maternal symptom load and mother-infant interaction, and prevalence of postpartum episodes

Table 4 presents the symptom load in the BD sample at 3 months postpartum and affective episodes during the 0–3 months postpartum period.

**Table 4:** Symptom load of BD mothers at 3 months postpartum and proportion with at least one affective episode during the 0–3 months postpartum period

	Total *N* = 26				Primiparous *N* = 13				Multiparous *N* = 13			
	n	%	M	range	n	%	M	range	n	%	M	range
Symptom load 3 months postpartum												
Euthymia												
IDS score 0–13	8	30.8	5.3	0–12	6	46.1	6.7	2–12	2	15.4	1.0	0–2
+ YMRS score 0–7			1.3	0–2			1.7	0–2			0	
Depressive symptomatology (IDS score)												
Mild (14–21)	6	23.1	18.3	16–21	3	23.1	18.7	18–19	3	23.1	18.0	16–21
Moderate (22–30)	5	19.2	26.2	23–29	1	7.7	29.0		4	30.8	25.5	23–29
Severe (31–38)	4	15.4	36.0	32–38	2	15.4	37.0	36–38	2	15.4	35.0	32–38
Manic symptomatology (YMRS score)												
Hypomania (8–20)	2	7.7	14.0	10–18	0				2	15.4	14.0	10–18
Mixed state												
Mild (IDS 14–21 + YMRS 8–20)	1^a^	3.8			1	7.7						
Affective episodes 0–3 months postpartum	12	46.1			4	30.8			8	61.5		
Depressive episode												
Mild	3	11.5			1	7.7			2	15.4		
Moderate	2	7.7			0				2	15.4		
Severe	4	15.4			3	23.1			1	7.7		
Manic episode	2	7.7			0				2	15.4		
Two subsequent episodes (hypomania; depression)	1	3.8			0				1	7.7		

*IDS* = Inventory of Depressive Symptomatology, *YMRS* = Young Mania Rating Scale

^a^IDS score = 14, and YMRS score = 11.5

At 3 months postpartum, 38.4% of the BD mothers had moderate to severe symptoms (34.6% depressive, 3.8% hypomanic), 30.7% had mild symptoms (23.1% depressive, 3.8% hypomanic and 3.8% mixed) and 30.8% of the BD mothers were euthymic. No significant associations were found between symptom load and interaction quality, either when treating symptom load as a continuous or categorical variable (Additional file 2 and Additional file 3). However, the association was of medium effect size (Cohen’s *d* 0.70) on subscale 1, when treating symptom load as a categorical variable.

During the first three postpartum months, 12 (46.1%) BD mothers were assessed to have had affective episodes, six (23.1%) experienced subthreshold symptoms, and eight (30.8%) were euthymic.

More multiparous than primiparous BD mothers had at least mild affective symptoms at 3 months postpartum (84.7% vs. 53.9%), and at least one postpartum illness episode (61.5% vs. 30.8%).

## Discussion

The present study addresses the knowledge gap on early mother-infant interaction in the context of maternal BD. Although there were individual variations, our results indicate significantly more concerns in mother-infant interactions at 3 months postpartum when the mother has BD compared to when the mother has no mental disorder. More concerns were observed in all three domains that were studied: maternal behaviour, infant behaviour and dyadic coordination. The results were not associated with concurrent maternal affective symptom load.

### Sample characteristics

Although the women in the non-clinical sample had a higher level of education and work participation, the BD sample was in no way characterised by social adversity. Two-thirds had managed to complete secondary school and 50% had an even higher level of education, which is on par with the Norwegian population. Approximately 60% were either in full- or part-time work. The significant group differences reflect the skewed resourcefulness of the comparison group, even in comparison with the general population of Norway.

The infants in our BD sample did not have poorer newborn status than the comparison infants. With the exclusion criteria, we eliminated the most severe neonatal outcomes (i.e., prematurity and serious medical conditions). Neither were the infants in our BD sample affected by the negative birth outcomes described in the literature (i.e., SGA and low birth weight) [16]. Thus, the infants in our BD sample represented low biomedical risk with regard to these factors.

### Mother-infant interaction

As anticipated, there were more concerns in the mother-infant interactions of the BD sample. Within the results, there are interesting nuances. On the one hand, the mothers in the BD sample resembled the non-clinical mothers in display of positive affect, visual contact and amount of verbalisation. However, in the BD sample, these maternal behaviours were less sensitively attuned and contingent on the infants’ signals. The decreased maternal sensitivity corresponds with observations and tendencies in prior studies [20–22]. Furthermore, our results align with previous findings of BD mothers vocalising more with their infants [21] and toddlers [37] than mothers with unipolar depression. Interestingly, when talking with their toddlers, a speech pattern with little turn-taking was representative of the mothers with BD, implying reduced sensitivity to child cues [37].

The mothers in our BD sample were not characterised by expressed negative affect, contrasting the findings of Hipwell et al. [21]. A possible explanation for the discrepant findings may be that their case group consisted of both mothers with BD and with unipolar depression. Although not conclusive, their analyses suggest that the unipolar depression group contributed the most to expressed negative affect [21]. Thus, when combining the results from the two maternal subscales, the mothers with BD were generally positive and friendly, but more BD mothers than comparison mothers displayed difficulties in different aspects of sensitivity, involvement and contingent responsiveness. Communication that is positive, but noncontingent, may have unintended consequences. Infants from 2 months of age are sensitive to the social contingency of maternal behaviour and respond to noncontingent communication with a decrease in their own positive affect [38, 39].

In fact, the infants in our BD sample were significantly less expressive of positive affect and happiness, as well as overall less expressive in communication and responsiveness, than the comparison infants. In combination with little expression of negative affect and irritability (subscale 4), a majority of the infants in the BD sample were quiet and subdued. This corroborates a trend reported by Hipwell et al. that infants in the study group were less expressive at 12 months postpartum than controls [21]. Notably, our observations of little expressed negative affect and dysregulation do not necessarily contradict the findings of Johnson et al. of disruptions in physiological stress responsivity and regulation [17]. The infants of BD mothers in their study did not differ from controls in overt display of negative affect or behaviour. Their maladaptive regulation patterns only showed on physiological measures (respiratory sinus arrhythmia, RSA) [17]. However, physiological stress responsivity and regulation have not been measured in the present study.

With reduced maternal sensitivity and contingent responsiveness (i.e., behaviours that promote reciprocity) and reduced communicative and responsive behaviours in the infants, the low level of dyadic coordination in our BD sample is a conceivable finding. Several mothers and infants had discrepant affective states, modest joint attention and minimal reciprocal communication, thus resulting in constricted dyadic exchanges. In other words, several mothers and infants did not seem to “find” and synchronise with each other. Whereas group differences in the present study are significant, the observed difficulties in mother-infant reciprocity in dyads of mothers with bipolar depression did not reach the level of significance in a previous study [22]. The authors discuss whether their measurements were not sensitive enough to capture subtle differences in small samples [22]. The PCERA has been found to have good sensitivity and discriminant validity [31, 32], even with small sample sizes [40].

There are alternative interpretations of the dyadic findings. One line of thought has been touched upon above that maternal interaction patterns of reduced sensitivity and noncontingency may prompt specific responses in the infants, i.e., the infant withdraws from interaction and displays little affect and communication [38, 39]. A second line of thought concerns what effect the infant’s behaviour may have on the mother. A small body of research highlights how infants’ characteristics in communicative behaviour may reinforce or diminish caregiver contingent responsiveness [41]. Are BD-offspring constitutionally less expressive and communicative as young infants, making them “difficult” dyadic partners? This is an unanswered and complex question concerning infants of mothers with inheritable mental disorders [6, 7, 23]. The most obvious factors to consider, such as birth outcomes and infant medication exposure, could not shed light on the infants’ behaviour in the present study. However, regardless of who contributes the most to the lack of reciprocity, dyadic difficulties are at risk of being maintained and strengthened by self-reinforcing mechanisms.

### Associations between maternal symptom load and mother-infant interaction

The fact that 38.4% in the BD sample had moderate to severe affective symptoms at 3 months postpartum and 46.1% had prior or ongoing postpartum episodes illustrates that many women with BD experience an immense personal burden when the mother-infant relationship is established. Most deviations were of depressive character. This is consistent with depression being the most commonly reported postpartum episode for women with BD [42, 43].

Intuitively, the presence of affective symptoms should reduce the quality of mothers’ interaction contributions. However, and contrary to our hypothesis, concurrent symptom load was not associated with interaction quality, neither when analysing individual subscales nor the PCERA scale as a whole. Thus, we did not observe a “dose-response” effect between current symptom load and interaction quality. We may remark that a type II error cannot be ruled out in our hypothesis testing. There was a medium to large effect size on subscale 1, when treating symptom load as a categorical variable (Additional file 2). Still, given the non-significant association, we treat our assumption as being too simplistic and straightforward. It is likely that interactional dynamics are influenced by several factors. First, studies of maternal depression have highlighted individual differences in overt maternal behaviour despite the same symptom level [44]. Second, there may be individual differences in how the infants experience and thus respond to the mothers’ symptoms [44]. Third, transient illness episodes have been found to be less influential on interaction quality than prolonged ones [44, 45]. In the women with BD, we observed large variations concerning the time of onset, duration, severity and consequences of mood episodes. The complexity and lack of statistical power did not allow us to explore this further.

Furthermore, there may be other factors at play related to having BD that may affect interaction quality regardless of symptom level. In several studies, persons with BD have been found to have deficits in emotion recognition and mentalising across different phases of illness [46, 47]. Sensitivity and contingent responsiveness in mother-infant interactions imply emotion recognition and mentalising capabilities especially with very young infants, who have small communicative repertoires and subtle emotional and social cues. Emotion recognition and mentalising were not investigated in the present study but may be important aspects to explore in future studies.

Altogether, the findings point to a need for clinical interventions that sensitise mothers to their infant’s cues on a micro-level, for example by using specialised approaches such as the Newborn Behavioral Observations System [48] and the PCERA. With its thorough interaction assessment, the PCERA is also valuable for clinical application [31, 49]. Through viewing recorded samples of interaction, mothers can get detailed feedback of their infant’s cues with guidance on contingent maternal behaviour. Such approaches may strengthen interactional reciprocity and synchronicity.

### Strengths and limitations

Whereas prior studies have primarily assessed maternal interaction behaviour, the main strength of the present study is the inclusion of both maternal and infant behaviour and their dyadic coordination. This allowed for a more comprehensive interaction analysis. Almost all interactions in the BD sample were carried out in their homes. This strengthens the ecological validity of the data [50]. Furthermore, the mothers (except one) confirmed the representativeness of the interaction sessions.

The coders were not naïve to the mothers’ BD status. This may have influenced their ratings. Counteracting such influences, the variables in PCERA are strictly operationalised in the manual, with extensive and precise descriptions regarding rating. Additionally, the main coder in the present study also coded the comparison data. In that study, group allocation was unknown to the coders.

The relative resourcefulness of the included dyads is likely to limit the generalisability of our findings to subgroups with similar characteristics. However, it is noteworthy that we nevertheless found interactional concerns and significant group differences. A higher level of interactional concerns may be observed in dyads of BD mothers with risk factors such as single motherhood, substance abuse, socioeconomic difficulties, and infants with negative birth outcomes. In the same ways that the BD sample is not representative of all dyads in which the mother has BD, the dyads of the comparison group are not representative of the general population. The comparison sample consists of mothers without known substance abuse or mental health problems, an above average level of education, and healthy infants without birth complications. Thus, it is likely that both samples are skewed towards the resourceful end of the populations they represent.

Given the relatively small sample size, the findings need to be interpreted with some caution. A small sample size increases the width of confidence intervals and limits generalisation.

Replication studies and studies of less resourceful dyads are needed. We also suggest studies of father-infant interaction when the mother has BD to investigate whether the low level of expressiveness we found in the infants may be relationship-specific.

## Conclusion

The present study identified concerns in maternal and infant interaction behaviour at 3 months postpartum in a relatively resourceful sample of mothers with BD and their infants compared to dyads with mothers without mental health problems. Most interactional concerns were identified in dyadic coordination. The findings were not influenced by the BD mothers’ concurrent affective symptoms.

To achieve dyadic coordination and synchrony, the interaction partners need to be familiar with each other’s behavioural repertoire and interaction rhythms [51]. Thus, we suggest interventions specifically focusing on sensitising and supporting mothers to read infants’ cues on a micro-level. This may help them to respond contingently and improve interactional reciprocity and synchronicity.

## Supplementary information

**Additional file 1.** Interaction score comparisons (mean) between groups (infants exposed vs. not exposed to BD medication) in BD sample (*n* = 26) on PCERA subscales.

**Additional file 2.** Interaction score comparisons (mean) between groups (maternal high vs. low symptom load) in BD sample (*n* = 26) on PCERA subscales.

**Additional file 3.** Correlations between PCERA subscales and symptom load in BD sample (*n* = 26).

## Data Availability

The datasets generated and analysed during the present study (film recordings) will not be shared or made publicly available, since participants may be identifiable. Request of permission to access data may be sent to the corresponding author.

## Abbreviations

BD: Bipolar disorder
EPDS: Edinburgh Postnatal Depression Scale
IDS: Inventory of Depressive Symptomatology
PCERA: Parent-Child Early Relational Assessment
YMRS: Young Mania Rating Scale
