# Supplementary material for: Mothers with and without bipolar disorder and their infants: group differences in mother-infant interaction patterns at three months postpartum
Source: BMC Psychiatry. 2019 Sep 18;19:292. doi: 10.1186/s12888-019-2275-4 (PMC6751750; doi:10.1186/s12888-019-2275-4)
Supplement: Supplementary file 1 — Additional file 1. Interaction score comparisons (mean) between groups (infants exposed vs. not exposed to BD medication) in BD sample (n = 26) on PCERA subscales. [file 12888_2019_2275_MOESM1_ESM.docx]

**Additional file 1.** Interaction score comparisons (mean) between groups (infants exposed vs. not exposed to BD medication) in BD sample (n=26) on PCERA subscales.

| **Subscale** | **Infants exposed to BD medication (n=17)**  Mean (sd) | **Infants not exposed to BD medication (n=9)**  Mean (sd) | **Mean difference**  95 % CI | **Sign.*** | **Cohen’s *d*** |
| --- | --- | --- | --- | --- | --- |
| S1-  Maternal positive affective involvement, sensitivity and responsiveness | 3.6 (0.45) | 3.8 (0.41) | 0.15 (-0.22 to 0.53) | 0.40 | 0.46 |
| S2-  Maternal negative affect and behaviour | 3.9 (0.40) | 4.1 (0.35) | 0.21 (-0.11 to 0.53) | 0.19 | 0.53 |
| S3-  Infant positive affect, communicative and social skills | 3.2 (0.68) | 3.2 (0.81) | -0.01 (-0.62 to 0.61) | 0.98 | 0.01 |
| S4-  Infant dysregulation and irritability | 4.0 (0.47) | 4.2 (0.44) | 0.18 (-0.22 to 0.57) | 0.36 | 0.44 |
| S5-  Dyadic mutuality and reciprocity | 2.6 (0.74) | 2.6 (1.03) | -0.09 (-0.81 to 0.63) | 0.80 | 0.10 |
| S6-  Dyadic tension | 3.6 (0.59) | 3.7 (0.41) | 0.04 (-0.41 to 0.50) | 0.85 | 0.20 |

*Independent sample t-test
