# Supplementary material for: Mothers with and without bipolar disorder and their infants: group differences in mother-infant interaction patterns at three months postpartum
Source: BMC Psychiatry. 2019 Sep 18;19:292. doi: 10.1186/s12888-019-2275-4 (PMC6751750; doi:10.1186/s12888-019-2275-4)
Supplement: Supplementary file 2 — Additional file 2. Interaction score comparisons (mean) between groups (maternal high vs. low symptom load) in BD sample (n = 26) on PCERA subscales. [file 12888_2019_2275_MOESM2_ESM.docx]

**Additional file 2.** Interaction score comparisons (mean) between groups (maternal high vs. low symptom load) in BD sample (n=26) on PCERA subscales.

| **Subscale** | **High symptom load (n=11)**  Mean (sd) | **Low symptom load (n=15)**  Mean (sd) | **Mean difference**  95 % CI | **Sign.*** | **Cohen’s *d*** |
| --- | --- | --- | --- | --- | --- |
| S1-  Maternal positive affective involvement, sensitivity and responsiveness | 3.5 (0.44) | 3.8 (0.42) | 0.22 (-0.13 to 0.57) | 0.21 | 0.70 |
| S2-  Maternal negative affect and behaviour | 3.9 (0.39) | 4.0 (0.39) | 0.11 (-0.21 to 0.43) | 0.47 | 0.26 |
| S3-  Infant positive affect, communicative and social skills | 3.1 (0.73) | 3.3 (0.71) | 0.18 (-0.40 to 0.77) | 0.52 | 0.28 |
| S4-  Infant dysregulation and irritability | 3.9 (0.48) | 4.1 (0.43) | 0.23 (-0.14 to 0.60) | 0.21 | 0.44 |
| S5-  Dyadic mutuality and reciprocity | 2.6 (0.78) | 2.6 (0.90) | 0.07 (-0.63 to 0.76) | 0.84 | 0.08 |
| S6-  Dyadic tension | 3.6 (0.62) | 3.7 (0.46) | 0.07 (-0.37 to 0.50) | 0.76 | 0.18 |

*Independent sample t-test
