# Supplementary material for: Mothers with and without bipolar disorder and their infants: group differences in mother-infant interaction patterns at three months postpartum
Source: BMC Psychiatry. 2019 Sep 18;19:292. doi: 10.1186/s12888-019-2275-4 (PMC6751750; doi:10.1186/s12888-019-2275-4)
Supplement: Supplementary file 3 — Additional file 3. Correlations between PCERA subscales and symptom load in BD sample (n = 26). [file 12888_2019_2275_MOESM3_ESM.docx]

**Additional file 3.** Correlations between PCERA subscales and symptom load in BD sample (n=26).

|  | Depression | | Hypomania/mania | |
| --- | --- | --- | --- | --- |
| **Subscale** | r* | Sign. | r* | Sign. |
| S1-  Maternal positive affective involvement, sensitivity and responsiveness | 0.10 | 0.62 | 0.01 | 0.97 |
| S2-  Maternal negative affect and behaviour | 0.02 | 0.92 | 0.05 | 0.80 |
| S3-  Infant positive affect, communicative and social skills | 0.09 | 0.68 | 0.17 | 0.42 |
| S4-  Infant dysregulation and irritability | 0.24 | 0.25 | 0.15 | 0.45 |
| S5-  Dyadic mutuality and reciprocity | 0.10 | 0.63 | 0.02 | 0.91 |
| S6-  Dyadic tension | 0.06 | 0.76 | 0.02 | 0.93 |

*Pearson’s correlation coefficient. P-values from linear regression analysis.
